# Incidence of Surgical Site Infection Despite Preoperative Cefazolin Administration in Total Knee Arthroplasty Patients: A Tertiary Hospital Experience

**DOI:** 10.7759/cureus.43912

**Published:** 2023-08-22

**Authors:** Zaher Mikwar, Bassam AlRajhi, Bakur W Saimaldaher, Ahmed Al-Magrabi, Abdullah Khoja, Amir Abushouk

**Affiliations:** 1 General Surgery, King Abdulaziz Medical City, Jeddah, SAU; 2 College of Medicine, King Saud Bin Abdulaziz University for Health Sciences, Jeddah, SAU

**Keywords:** saudi arabia, tertiary hospital, total knee replacement, cefazolin, surgical site infection

## Abstract

Introduction: Surgical site infection (SSI) is an infection that occurs after surgery on the incision site. Cefazolin is an old-generation antibiotic that decreases the risk of SSI. This study aimed to determine the relationship between the incidence of SSI, cefazolin administration, and the factors contributing to the relationship between them.

Methods: This is a retrospective study that used a data collection sheet to collect variables from the medical records of patients who underwent total knee arthroplasty (TKA) between 2016 and 2020. We looked mainly for the type of antibiotics given preoperatively, the number of doses given, discontinuation of antibiotics postoperatively, length of hospital stay, intensive care unit (ICU) admission, and SSI occurrence.

Results: A total of 195 patients were included. The majority (87.8%) were given two grams of cefazolin. Patients who have taken one gram of cefazolin had a slightly higher hospital stay than those who took two grams. However, all the patients did not develop an SSI.

Conclusion: There was no incidence of SSI despite preoperative cefazolin administration in TKA patients. Patients who received higher doses of cefazolin had a shorter length of hospital stay.

## Introduction

Surgical site infection (SSI) is defined as an infection that occurs at the incision site within the first 30 days of surgery or a year of foreign body implantation [1]. It is considered one of the most common and crucial postoperative complications because it can lengthen patients’ hospital stays after surgery, compromise patients’ health, and deplete hospital financial resources [2,3]. In Europe, according to the annual epidemiological report of the European Center of Disease Control (ECDC) in 2017, which included 13 different European countries, 648,512 surgical procedures were performed in 1639 reported hospitals with 10,149 SSI cases reported [4]. SSI has many possible risk factors that predispose patients to undergo surgical procedures to develop an infection, such as age, diabetes, nutritional status, obesity, smoking, duration of the surgery, and prepping before the procedure [1,3,5]. Due to these risk factors, SSI prevention and control remain challenging in the surgical field [6]. Despite new SSI preventive measures and strategies, prophylactic antibiotics remain the principal preventive methods [7]. SSI is mainly preventable using preoperative antibiotics, which are antimicrobial agents administered before surgery to prevent bacterial infections. The most commonly used preoperative antibiotics are narrow-spectrum and old-generation drugs, such as cefazolin and cefuroxime [8].

Modern guidelines suggest the use of cefazolin as the primary prophylactic antibiotic because of its favorable safety profile, low cost, and targeted activity against microorganisms [9]. In a recent retrospective cohort study that involved data from 862,918 patients who underwent total knee arthroplasty (TKA), SSI cases were recorded to be 0.22% in patients who underwent TKA. Cefazolin was the most frequently used prophylactic antibiotic in that study (74.1%) [7]. Furthermore, the same study revealed that a higher incidence of SSI was associated with the use of antibiotic regimens other than cefazolin in TKA patients [7]. Most surgical prophylaxis guidelines, including the collective guideline for surgical prophylaxis and the Australian Medicines Handbook, propose increasing the dose of prophylactic cefazolin dose to three grams in patients weighing 120 kg or more because obese patients are more susceptible to health-related issues and are more likely to undergo different types of surgeries. Moreover, a retrospective case-control study was conducted on patients who received two grams of cefazolin before elective surgeries and were divided into obese and non-obese groups. Although not statistically significant, the prevalence of SSI among obese patients was almost twice as high as that among non-obese patients [10]. Similar results have been reported in another study [11].

In Saudi Arabia, an observational prospective study of 1611 patients revealed an SSI incidence rate of 11.4% [12]. In another study conducted locally, there was a remarkable decrease in SSI cases in King Fahad Hospital, from 20 cases per 1000 operations in 2009 to 3.5 cases per 1000 operations in 2018. This significant decline is attributed to the application of a hospital accreditation system that helped assess the hospital's performance and enhance the quality of care provided by the hospital [1,13]. Modern guidelines recommend administering preoperative antibiotics within an hour before the incision because the risk of SSI increases if preoperative antibiotics are administered more than two hours before the incision [14]. However, this has not been heavily supported and remains open to debate. Furthermore, the duration and discontinuation of preoperative antibiotic administration after surgery remain unclear [7].

This study aimed to determine the association between the incidence of SSI and cefazolin administration and the factors contributing to this relationship. The significance of this study was to determine the infection rate despite following surgical guidelines, including preoperative administration of cefazolin in King Abdulaziz Medical City (KAMC) for the National Guard, Western Region, Jeddah, Saudi Arabia.

## Materials and methods

Study design and settings

This retrospective cohort study was conducted at King Abdulaziz Medical City (KAMC) in Jeddah, Saudi Arabia. Data were collected from the BestCare system, which contains the medical files of every patient in KAMC. We reviewed patient files that included elective or semi-elective cases that underwent TKA at KAMC between 2016 and 2020. The inclusion criteria were as follows: (1) patients aged 18 to 70 years; (2) administration of cefazolin preoperatively; and (3) TKA whether unilateral, bilateral, or both; (4) A total of 623 patients were included in this study. All emergency cases were excluded from this study. Emergency cases were defined as patients who underwent TKA due to a motor vehicle accident, fall, or any traumatic incident rather than undergoing the surgery electively. Using the Raosoft online sample size calculator, it was recommended to use a sample size of 238 patients, considering the following: margin of error, 5%; confidence interval, 95%; and approximated population size, 623. A consecutive sampling method was used.

Data collection

The SSI bundle offered by the infectious unit in KAMC, comprising 16 variables, was used as a data collection form to obtain data from the BestCare System. The data were manually entered into a Google Forms webpage for further analysis. The data collected mainly included variables such as length of hospital stay, preoperative cefazolin dose, number of doses administered, prophylactic antibiotic discontinuation within 24 h postoperatively, SSI occurrence, SSI form if it occurred, and intensive care unit (ICU) admission. Medical records were collected only to double-check the availability of missing variables and were discarded before data analysis. Only the lead author had access to data to preserve patient anonymity. This study was approved by the Institutional Review Board (IRB) of King Abdullah International Medical Research Center (KAIMRC) (Study Number: SP21J/126/03).

Data analysis

Categorical variables were presented as frequencies and percentages, and continuous variables were presented as means and standard deviations. The chi-square test was used for categorical variables, and the t-test was used for continuous variables. Statistical significance was set at p<0.05. The data were analyzed using John's Macintosh Project (JMP) software version 10.0 (SAS Institute Inc., Cary, NC, USA). A consent form was not needed because the study utilized a chart review for data collection.

## Results

A total of 195 patients who underwent TKA and were given cefazolin preoperatively were included. Most of the patients were female (78.97%), non-smokers (96.37%), and approximately 43% had diabetes (Table 1). The minimum length of hospital stay was three days, and the maximum was 23 days. Most patients were discharged within the first eight days of admission. Postsurgical prophylactic antibiotics were continued beyond 24 h in 95 patients (48.72%). During their hospital stay, only two patients were admitted to the ICU. Before surgery, 170 (87.18%) patients were administered two grams of cefazolin. Additionally, patients who have taken one gram of cefazolin had a slightly longer hospital stay than those who took two grams of cefazolin, which was statistically significant with a p-value of 0.0374 (Table 2). One patient was reported to have a high fever four days after surgery. A culture was ordered to check for infection since this is part of the hospital guidelines to order a culture if a fever persists more than three days following a TKA, but there was no growth in the culture. None of the patients who were administered cefazolin (one or two grams) developed any SSI after the surgery.

**Table 1:** Factors affected by cefazolin administration and/or SSI occurrence ICU, intensive care unit; SSI, surgical site infection *One dose is the equivalent of one gram of cefazolin

	*Doses of cefazolin given		P-value
Characteristic	One	Two	-
Length of hospital stay (days) (mean±SD)	8.96±3.51	7.34±2.98	0.0374*
Discontinuation of prophylactic antibiotics within 24 hours	0 (0.00%)	1 (1.04%)	0.7318
ICU admission	0 (0.00%)	1 (0.51%)	0.7006
SSI occurrence	0 (0.00%)	0 (0.00%)	-

**Table 2:** Demographic characteristics of study participants *One dose is the equivalent to one gram of cefazolin

Characteristics	*One dose of cefazolin	Two doses of cefazolin	P-value
N=195	25	170	-
Gender			
Male	7 (3.59%)	34 (17.44%)	0.3594
Female	18 (9.23%)	136 (69.74%)	
Age (years) (mean±SD)	61.56±5.49 years	61.44±5.41 years	0.9218
Weight (mean±SD)	86.08±9.83	86.44±15.41	0.8772
Height (mean±SD)	154.01±7.51	156.20±9.50	0.1976
Smoker	2 (1.04%)	5 (2.59%)	0.1875
Diabetic	14 (7.18%)	70 (35.90%)	0.1623

## Discussion

In this study, none of the patients who were administered cefazolin (N=195) before surgery developed any surgical infection at the incision site. Prevention of SSI is important for preserving patient health, decreasing the length of hospital stay, and decreasing the consumption of financial resources. This study showed that administering a higher dose of cefazolin is associated with a shorter hospital stay for patients who underwent TKA.

The low incidence of SSIs in patients who underwent orthopedic surgery and were administered cefazolin has been reported elsewhere in the literature [7,15,16]. In one study conducted in Saudi Arabia, no patients developed SSI (SSI rate = 0) during anterior cruciate ligament reconstruction surgeries, an orthopedic surgery performed on the knee, and cefazolin was administered preoperatively [17]. In a study conducted by Zastrow et al., antibiotic prophylaxis regimens other than cefazolin were associated with an increased SSI risk [7]. These results are significant because they indicate the effectiveness of cefazolin in preventing surgical infection. In comparison, Sommerstein et al. stated that using a double dose of cefuroxime only minimizes the incidence of SSI in patients weighing less than 80 kg, which is inconsistent [18].

Wyles C et al. concluded that a single dose of perioperative antibiotics, typically cefazolin, does not increase the risk of SSI in unicompartmental knee arthroplasty (UKA) in comparison with 24-hour IV antibiotics [16]. In a previous study, two of 296 patients developed SSI [16]. Due to the relatively small sample size and low incidence rate of SSI in TKA, these results may not be generalizable to other populations. However, Morris et al. concluded that higher SSI rates were associated with cefazolin underdosing, which was defined as the use of one gram of cefazolin in patients weighing 80 kg or more, or a cefazolin dose of less than three grams in patients weighing 120 kg or more [11]. Although there was some inconsistency between double dosing of preoperative prophylaxis and SSI in patients weighing 80 kg or more in one study [18], the prevalence of SSI in obese patients was almost twice as high as that in non-obese patients in other studies [10,11].

Since joint arthroplasty surgeries, such as TKA and UKA, tend toward shorter hospital stays, the results of our study suggest that administering higher doses of cefazolin, especially to obese individuals, may decrease the hospital stay, morbidity, and mortality of patients undergoing TKA. Due to the low cost, safety, and efficacy of cefazolin, the results of our study suggest that two grams of cefazolin is the standardized preoperative antibiotic dose for all adult patients undergoing TKA. Morris et al. stated that the cost of giving higher doses of cefazolin to patients weighing 80 kg or more to prevent one SSI is cost-effective which may be applicable in Saudi Arabia [11].

This study has several limitations. First, only a small sample size was obtained from a single hospital. We attempted to minimize this by including all patients who underwent TKA between 2016 and 2020 and met our inclusion criteria. Second, the original data collection form included two variables, the length of surgery and postoperative temperature, but they were removed during the data collection process because they were missing in most of the medical records. Third, the comparison between the two groups may not be ideal since the one-gram group had 25 patients while the two-grams group had 170 patients. Fourth, because the data were collected manually from electronic medical records, underreporting and data entry inaccuracies can be suspected.

## Conclusions

SSI is a clinically significant complication of all types of surgeries. In our study, there was no incidence of SSIs in any of the patients who were administered cefazolin and then underwent TKA. These results are crucial for orthopedic surgeons to consider increasing the preoperative cefazolin dose in obese and non-obese patients undergoing TKA. Further studies are needed to determine the efficacy of other types of antibiotics (e.g., cefuroxime) in preventing SSI in other orthopedic surgeries such as total hip arthroplasty and total shoulder arthroplasty.
